# A ketogenic diet normalizes interictal cortical but not subcortical responsivity in migraineurs

**DOI:** 10.1186/s12883-019-1351-1

**Published:** 2019-06-22

**Authors:** Cherubino Di Lorenzo, Gianluca Coppola, Martina Bracaglia, Davide Di Lenola, Giulio Sirianni, Paolo Rossi, Giorgio Di Lorenzo, Vincenzo Parisi, Mariano Serrao, Mackenzie C. Cervenka, Francesco Pierelli

**Affiliations:** 1 IRCCS – Fondazione Don Carlo Gnocchi, Milan, Italy; 2 Department of Medico-Surgical Sciences and Biotechnologies, “Sapienza” University Rof Rome Polo Pontino, Latina, Italy; 3 Dietary Section, Associazione Eupraxia, Rome, Italy; 4 INI, Headache Clinic, Grottaferrata (RM), Italy; 5 Laboratory of Psychophysiology, Department of Systems Medicine, University of Rome “Tor Vergata”, Rome, Italy; 6 Research Unit of Neurophysiology of Vision and Neurophthalmology, IRCCS – Fondazione Bietti, Rome, Italy; 7 Department of Neurology, Johns Hopkins University School of Medicine, Baltimore, USA; 8 IRCCS – Neuromed, Pozzilli, IS Italy

**Keywords:** Ketogenesis, Ketogenic diet, Habituation, Migraine, Nociceptive blink reflex, Pain-related evoked potentials

## Abstract

**Background:**

A short ketogenic diet (KD) treatment can prevent migraine attacks and correct excessive cortical response. Here, we aim to prove if the KD-related changes of cortical excitability are primarily due to cerebral cortex activity or are modulated by the brainstem.

**Methods:**

Through the stimulation of the right supraorbital division of the trigeminal nerve, we concurrently interictally recorded the nociceptive blink reflex (nBR) and the pain-related evoked potentials (PREP) in 18 migraineurs patients without aura before and after 1-month on KD, while in metabolic ketosis. nBR and PREP reflect distinct brain structures activation: the brainstem and the cerebral cortex respectively. We estimated nBR R2 component area-under-the-curve as well as PREP amplitude habituation as the slope pof the linear regression between the 1st and the 2nd block of 5 averaged responses.

**Results:**

Following 1-month on KD, the mean number of attacks and headache duration reduced significantly. Moreover, KD significantly normalized the interictal PREP habituation (pre: + 1.8, post: − 9.1, *p* = 0.012), while nBR deficit of habituation did not change.

**Conclusions:**

The positive clinical effects we observed in a population of migraineurs by a 1-month KD treatment coexists with a normalization at the cortical level, not in the brainstem, of the typical interictal deficit of habituation. These findings suggest that the cerebral cortex may be the primary site of KD-related modulation.

**Trial registration:**

ClinicalTrials.gov NCT03775252 (retrospectively registered, December 09, 2018).

## Background

Migraine causes the highest rate of headache-related disability and is one of the most prevalent non-fatal diseases with the third highest disease burden worldwide [1]. Despite great efforts to understand the pathophysiology of migraine and other primary headache disorders, the efficacy of current prophylactic treatments is often unsatisfactory because of the low responder rates (approximately half of patients) and adverse effects prompting cessation of therapy [2]. Therefore, innovative, more tolerated and effective treatments are needed (such as recently studied anti CGRP antibodies [3]), and there is a critical need for non-pharmacological treatments [4, 5] that in many cases have specific pathophysiological targets [4–6]. Among these treatments, dietetic interventions play a relevant role. One of the most promising dietetic approaches is the so-called ketogenic diet (KD), characterised by a high fat and a low carbohydrate intake that induces a state of metabolic ketosis, i.e. hepatocytes conversion of fatty acids to ketone bodies (KBs) [7]. In recent years, our group observed a possible effect of a weight-loss (calorie-restricted) KD for migraine prophylaxis, consistent with a previous report [8]. In fact, we retrospectively described the case of a pair of twin sisters whose migraines improved during their weight-loss KD [9]. In subsequent prospective proof of concept studies, we confirmed our previous observation in migraineurs [10] and patients with chronic cluster headaches [11]. A possible explanation for this outcome could involve the concomitant treatment of comorbid metabolic conditions, such as obesity [12, 13], and insulin resistance [14, 15] that are described in migraineurs and are both improved by KDs that induce weight loss. However, the mechanism of action of KD cannot be limited to treatment of the metabolic syndrome, particularly based on the observation that migraineurs without metabolic syndrome can also benefit. In addition, KD has been shown to be effective in other neurologic diseases through several other proposed mechanisms. For instance, the use of fasting for epilepsy dates back further than pharmacologic interventions and the KD which is designed to mimic fasting while maintaining adequate caloric intake is currently regarded as one of the preferred therapies for patients with drug resistant epilepsy [16], possibly caused by a diet-induced KB production causing modulation of cortical excitability [17, 18]. Aiming to understand mechanisms of KD clinical efficacy in migraine, we analysed the changes in cortical evoked potentials (EPs) in response to repeated visual and somatosensorial stimuli before and after 1-month on KD, during metabolic ketosis, and we observed a normalization of the baseline interictal deficit of habituation for both sensory modalities [19]. Whether the primary mechanism of action of KD in improving clinical and neurophysiological effects of migraine is solely based on alteration of activity in the cerebral cortex or the monoaminergic modulation of the nociceptive brainstem trigeminal system, which has been shown to play a pivotal role in the ignition of the migraine attack [20], remains to be determined.

The aim of this study thus is to investigate the function of the trigeminal system at the brainstem and cortical levels simultaneously in a group of migraineurs before and during KD, prospectively. To do so, we simultaneously recorded nociceptive blink reflex (nBR) and cortical pain-related evoked potentials (PREP) elicited by the same supraorbital painful stimuli [21–23] before and during metabolic ketosis induced by KD, in a group of patients with episodic migraine without aura, between attacks.

## Methods

### Subjects

We enrolled 22 consecutive patients with episodic migraine without aura who attended the headache centre of Sapienza University of Rome – Polo Pontino between 2016 and 2017. The primary inclusion criterion was being attack-free for at least 3 days before and after the recording sessions as determined by collecting headache diaries and telephone or e-mail interviews. Exclusion criteria were regular medication intake (i.e. antibiotics, corticosteroids, antidepressants, benzodiazepines, prophylactic migraine drugs) except for contraceptive pills. Other exclusion criteria included history of other neurological diseases, systemic hypertension, diabetes or other metabolic disorders, connective or autoimmune diseases, and any other type of primary or secondary headache. Female participants were always recorded mid-cycle. All study participants were naïve to the study procedure, received a complete description of the study, and provided written informed consent. The recordings of 4 patients were excluded from the statistical analysis because it was later determined that they were not free from migraine for at least 3 days before and after the recording session (see below). The final dataset thus comprises a group of 18 patients with migraine without aura (the diagnoses were made according to the ICHD-III Beta criteria, and further re-evaluated in all cases according to ICHD-III, code 1.1). At the time of the screening visit, we collected clinical features using up to two-month headache diaries as baseline and extracted the following information: the duration of migraines (years), attack frequency (migraines/month), attack duration (hours), and severity of migraine headache (0 = no severity; 1 = mild severity; 2 = partial severity; 3 = severe) and pain intensity (0–10 on the visual analogue scale, VAS). Before the KD intervention, the mean body mass index (BMI) of the enrolled migraineurs was 26.7 ± 4.6 kg/m^2^. After screenings for exclusion criteria, our control group consisted of 18 age- and gender- matched (one by one) healthy volunteers (HV; Table 1), with comparable BMIs (26.2 ± 4.5 kg/m^2^), we recruited among medical school students and healthcare professionals with no history of migraine or other neurologic disorders, who underwent the simultaneous recording of nBR and PREP.

**Table 1:** Baseline clinical and demographic characteristics of the study participants

Characteristics	HV (*n* = 18)	M-tot (*n* = 18)	
Women (n)	16	16	
Age (years)	39.7 ± 10.1	40.8 ± 11.7	
Duration of migraine history (years)		21.0 ± 11.5	
		Before	After 1-month KD
Attack frequency/month (n)		4.7 ± 2.5	1.9 ± 2.1 **
Attack duration (hours)		42.8 ± 24.0	17.9 ± 20.6 *
Disability (0–3)		2.7 ± 0.5	1.4 ± 1.1 **
Days since the last attack (n)		9.1 ± 8.5	12.4 ± 11.0
Body mass index (BMI)	26.2 ± 4.5	26.7 ± 4.6	25.3 ± 4.6

Data are expressed as means ± SD. Disability is a scale of self-perceived headache related disability (0= no disability; 1= mild disability; 2= partial disability; 3= total disability). * = *p* < 0.01 ** = *p* < 0.001 before vs. after 1-month ketogenic diet. *HV* Healthy Volunteers, *M-tot* Total of Migraine patients

### Ketogenic diet

Patients were administered by a dietician (Giulio Sirianni) skilled in KD training a 4-week normo-caloric ketogenic diet regimen (modified Atkins diet (MAD)) [7] consisting of low carbohydrate (approximately 15 g/day net carbohydrate intake; subtracting fiber grams), normal protein (about 0.7–1 g/Kg/day) and high fat (ranging from 120 to 170 g/day; Ketogenic Ratio (fat: carbohydrates and protein combined in grams) ranging 1.7–2: 1) from meals prepared using common foods. If necessary, to respect the caloric needs, these were supplemented with lipids in the form of a powder composed of medium chain triglycerides as well as omega-3 and long chain triglycerides (Ketoneural Lipid Complex, Medi-Diet s.r.l., Aprilia, Italy) with nutraceutical integrators (see also Table 1 in ref. #16 [19]). Each patient consumed lipid supplements, up to 30 g/day (omega-3, up to 1.65 g/day), according to the assigned daily nutritional recommendations. A daily urine dip stick test (Ketur Test, Roche Diagnostics, Monza, Italy) confirmed the presence of urinary KBs. The Ketur Test is able to detect the presence of urinary acetoacetate by the coloring of a label for values of KBs approximately > 0,5 mmol/l; according to KBs concentration, color blocks were reported as + (0.5–4 mmol/l), ++ (4–10 mmol/l), or +++ (> 10 mmol/l). Patients recorded the results in a headache diary along with meals, daily weight, possible adverse events, or side effects. Patients had medical supervision and laboratory blood tests (alanine aminotransferase, aspartate aminotransferase, gamma glutamic transpeptidase, lactic dehydrogenase, alkaline phosphatase, bilirubin, blood urea nitrogen, and creatinine) at the start and the end of the 4-week KD.

### Data acquisition

#### Simultaneous recording of nociceptive blink reflex (nBR) and pain-related evoked potentials (PREP)

The Simultaneous recording of nociceptive Blink Reflex (nBR) and Pain-Related Evoked Potentials (PREP) was performed in all subjects enrolled according to methods described elsewhere [22].

**Fig. 1:**
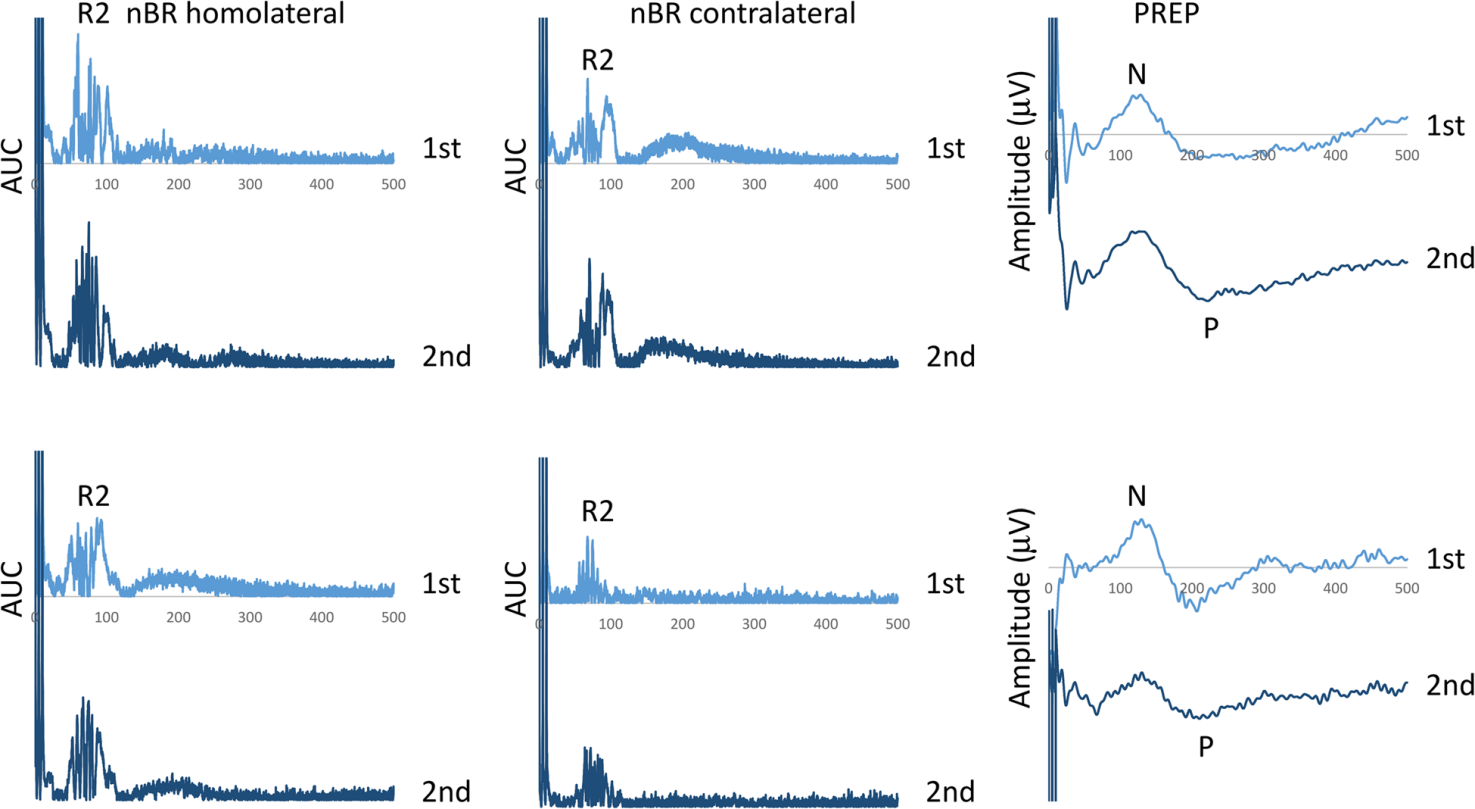
Explicative block recordings of nociceptive blink reflex (nBR) elicited homolaterally and contralaterally to the stimulated side, and pain-related evoked potentials (PREP) in a patient with migraine recorded before [upper panel] and after a 1-month ketogenic diet, during metabolic ketosis [lower panel]

In brief, a train of three pulses of galvanic stimuli (0.1-ms duration each, interpulse interval of 5 ms) was pseudorandomly (interstimulus intervals between 30- and 35-s) delivered at a fixed intensity of 1.2 × PT, via a stimulating electrode only applied to the right supraorbital notch, since our MO patients showed mono/bilateral migraine headaches not always localized on one specific side. In order to study habituation to nociceptive stimulations, we choose to acquire two blocks of six rectified electromyogram (homolateral and contralateral nBR) and electrocortical (PREP) responses with an interblock interval of 2 min. For each sweep, the post-stimulus period was recorded for 500 ms and subsequently off-line filtered (high-pass 10 Hz for nBR and low-pass 100 Hz for PREP). Each block was composed of five averaged responses (Fig. 1), as the first sweep was excluded from the signal analysis to avoid contamination with startle responses. For each averaged block, the R2 component of the area under the curve (AUC; μV x ms) calculated between 27 and 87 ms [24, 25] and the negative (N) and positive (P) vertex peck components were measured off-line by one investigator. Habituation of the nBR R2 component and of the N-P PREP amplitude were defined as the slope of the linear regression (calculated using the SLOPE function in Microsoft Excel) of the R2 AUC and of the N-P PREP amplitude between the first and the second blocks of recordings.

### Procedure

nBR and PREP recordings were simultaneously recorded during a single session including baseline (time 0) and 1-month after, during metabolic ketosis (> 0.5 mmol/l), as verified through urine ketone testing (Ketur Test), on the same day of the recording session. All recordings were performed in the afternoon (between 2:00 and 6:00 p.m.) by the same investigators (Martina Bracaglia and Davide Di Lenola) who were blinded to the whether recordings were obtained pre- or post-treatment and whether subjects were migraineurs or healthy volunteers. tThese investigators also did not meet the participants prior to the examination. All recordings were numbered anonymously and analysed off-line by one investigator (Gianluca Coppola), who was blinded to the subjects’ identities, but not blinded to the order of the blocks.

### Statistical analysis

Data were analysed in a blinded fashion by a single investigator (Vincenzo Parisi) using Statistica for Windows (StatSoft Inc., Tulsa, USA) version 8.0 software. Based on our previous study on the influence of KD on visual and somatosensory evoked potentials [19] we set our sample size to eighteen subjects which adequately powered the study to show statistically different results between controls and migraine participants if present. Both nBR and PREP components showed normal distribution at the Kolmogorov-Smirnov test. We used a General Linear Model approach to analyse the “between-factor” × “within-factors” interaction effect. The between-subject factor was “group” (HV vs. MO) or “time” (before vs. after KD) and within-subject factor was “blocks”. Three separate models of repeated measure ANOVA (rm-ANOVA) were performed, two for nBR (homolateral and contralateral to the stimulated side) and one for PREP. A regression analysis was used to disclose linear trends in nBR R2 AUC and N-P amplitude across the two blocks (slope) in each group. For slope, we employed ANOVA with group factor “group” (HV vs. MO) and Paired-sample t tests with group factor “time” (before vs. after KD).

Paired-sample t tests were used to compare the clinical data before vs. after KD. *P* values less than 0.05 were considered to indicate statistical significance.

Pearson’s correlation test was used to search for correlations among nBR and PREP slopes and clinical variables (duration of migraine history [years], attack frequency [n/month], attack duration [h/month], intensity of migraine headache [0–10], and migraine severity [0–3]).

## Results

### Clinical characteristics

The clinical characteristics of migraine patients before and after 1-month of KD are shown in Table 1. We observed a significant reduction in attack frequency (*t* = 5.27, *p* < 0.001), attack duration (*t* = 4.12, *p* = 0.001), and disability of headache attacks (*t* = 5.17, *p* < 0.001) after 1-month duration KD compared to baseline. BMI remained stable after KD.

### Basic neurophysiological parameters

We obtained measurable nBR and PREP recordings from all study participants (Fig. 1). Basic neurophysiological parameters (ST, PT, R2 nBR onset, N and P PREP latencies, see Table 2) were not significantly different between migraineurs and control participants (*P* > 0.05). In comparison with controls, 1st R2 component AUC was significantly lower in migraine on both homolateral (F_1,34_ = 18.682, *p* = 0.0001) and contralateral sides (F_1,34_ = 7.792, *p* = 0.008), while 1st N-P PREP amplitude block did not differ between groups (F_1,34_ = 2.194, *p* = 0.148).

**Table 2:** Electrophysiological parameters in healthy controls, and in the group of migraine patients before and after 1-month KD

	HV	KD Before	KD After
ST	3.1 ± 4.6	2.5 ± 1.2	2.5 ± 1.5
PT	14.1 ± 5.6	10.5 ± 4.6	11.1 ± 5.6
VAS (0–10)	7.0 ± 2.1	7.1 ± 1.8	6.9 ± 1.2
*Nociceptive Blink Reflex (nBR)*			
Homolateral R2 onset (ms)	32 ± 4.4	33.9 ± 7.9	32.9 ± 6.4
Homolateral R2 1st AUC block (μV x ms)	1.2 ± 0.6	0.5 ± 0.3 §	0.6 ± 0.3 §
Homolateral R2 slope	- 0.15 ± 0.11	0.02 ± 0.18 §	0.02 ± 0.17 §
Contralateral R2 onset (ms)	33.3 ± 4.6	33.7 ± 7.7	33.0 ± 5.9
Contralateral R2 1st AUC block (μV x ms)	1.1 ± 0.7	0.5 ± 0.4 §	0.5 ± 0.3 §
Contralateral R2 slope	- 0.17 ± 0.37	0.005 ± 0.17	0.02 ± 0.11
*Pain-related evoked potentials (PREP)*			
N (ms)	124.4 ± 16.3	127.0 ± 12.0	131.8 ± 18.0
P (ms)	241.2 ± 59.1	218.3 ± 30.1	224.5 ± 26.8
N-P 1st amplitude block (μV)	50.9 ± 28.5	39.1 ± 18.1	46.1 ± 19.1
N-P amplitude slope	- 6.9 ± 8.0	2.9 ± 11.0 §	- 9.1 ± 15.5 *

Data are expressed as means ± SD. * = *p* ≤ 0.01 before vs. after 1-month KD. § = *p* < 0.01 vs. HV

Lack of response habituation to repetitive noxious supraorbital stimulations characterized migraine group before KD intervention. This was confirmed for nBR R2 component homolateral to the stimulated side and for N-P vertex complex of PREP, but not for nBR R2 component recorded contralaterally to the stimulated side. In fact, in the rm-ANOVA model with nBR R2 AUC (homolateral) or N-P peak-peak amplitudes as dependent variable, multivariate test was significant for the “group” × “blocks” interaction effect (F_1,34_ = 11.973, *p* = 0.001 for nBR R2 homolateral; F_1,34_ = 9.420, *p* = 0.004 for PREP), but this was not the case in the model with nBR R2 AUC contralateral to the stimulated side (F_1,34_ = 3.456, *p* = 0.07). These data were confirmed by the linear regression slope which differed significantly between the two groups for nBR R2 AUC homolateral and N-P PREP amplitude over the 2 blocks (F_1,34_ = 12.081, *p* = 0.001 for nBR R2 homolateral; F_1,34_ = 6.613, *p* = 0.015 for PREP), but not for nBR R2 AUC contralateral (F_1,34_ = 3.467, *p* = 0.07).

Before KD intervention, the N-P amplitude slope correlated positively with the pain intensity during migraine headache as assessed by VAS (r = 0.471, *p* = 0.048) and with the duration of migraine history (r = 0.538, *p* = 0.021). In turn, VAS correlated positively with the duration of migraine (r = 0.472, *p* = 0.048) and with disability related to migraine (r = 0.513, *p* = 0.029). There were no other significant correlations between neurophysiological and clinical data.

### Ketogenic diet effects

Basic neurophysiological parameters (ST, PT, R2 nBR onset, N and P PREP latencies, see Table 2) were not significantly different before and after KD in migraineurs (*P* > 0.05).

**Fig. 2:**
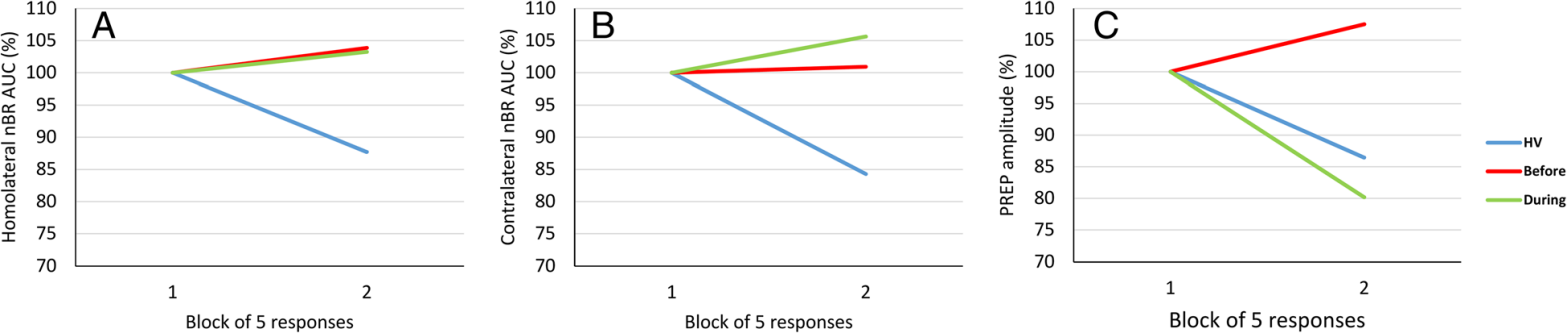
Habituation of the nociceptive blink reflex (nBR) R2 component area-under-the-curve (AUC) [**a** homolateral; **b** contralateral to the stimulated side] and pain-related evoked potentials (PREP) N-P amplitude [**c**] slopes in healthy controls and migraine patients before and after a 1-month ketogenic diet, during metabolic ketosis, in two blocks of five averaged responses expressed as a percentage of the first block

In the rm-ANOVA model with nBR R2 AUC homolateral or contralateral to the stimulated side as the dependent variable, the multivariate test did not reach the significance level for the “time” × “block” interaction effect (F_1,34_ = 0.0001, *p* = 0.991 for homolateral; F_1,34_ = 0.226, *p* = 0.637, for contralateral, Fig. 2).

Whereas, in the rm-ANOVA model using PREP N-P peak-peak block amplitude as dependent variable, the multivariate test was significant for the “time” × “block” interaction effect (F_1,34_ = 7.234, *p* = 0.011). The linear regression N-P slope of PREP amplitudes over all blocks differed significantly before compared to during KD (*t* = 2.817, *p* = 0.012, Fig. 2). Rm-ANOVA post-hoc analysis showed that 1st N-P PREP amplitude block did not change after 1-month KD.

## Discussion

We can summarize the most striking results of our study as follows: (a) HV exhibited a physiologic habituation in N-P amplitude slope of PREP, and both in homolateral and contralateral nBR; otherwise, migraineurs exhibited an interictal deficit of habituation for nBR, as expected [24], and for the PREP; (b) the lack of habituation observed before the diet is still present during ketosis for the nBR, but is normalized for the PREP. To the best of our knowledge, this is the first study in which there is a simultaneous recording of nBR and PREP, in order to assess the relationship between both evoked responses habituation and headache clinical features.

The most relevant finding of our study is that the response to trigeminal PREP habituates during the KD, while the simultaneous recording of nBR elicited by the same trigeminal nociceptive stimuli does not habituate. The phenomenon of habituation is the “response decrement as a result of repeated stimulation” and regarded as a basic form of learning and memory [26]. Habituation is due to changes in neuronal excitability that reflects changes in the adaptive short- and long- term neural plasticity phenomena and is determined by underlying genetic characteristics [27–29]. The described dissociation of habituation between PREP and nBR during metabolic ketosis reflects the different origins of the elicited responses to the repetitive nociceptive stimuli: the origin of PREP signal, although partially influenced by the brainstem, originates from cortical activity, whereas nBR is a pure brainstem response, not influenced by cortical activity. In fact, even if the expression of trigeminal PREP is obviously influenced by subcortical structures (nerve root, brainstem and thalamus) the most prominent component of trigeminal PREPs is generated by cerebral cortex [22]. and in particular the anterior cingulate cortex [30]. The emotional processing related to pain strongly influences the genesis of PREPs [31] possibly by modifying the balance between GABA and glutamate neurotransmission at the cortical level [32]. The results of our present study suggest that PREP habituation deficits have a direct relationship with increased migraine severity. Lack of PREP habituation worsens progressively with the subjective perception of severity of migraine and with the duration of migraine. Recent studies show that neural plasticity related to cortical pain processing can be influenced by brain derived neurotrophic factor (BDNF) [28]. In healthy humans, the habituation of PREP is negatively influenced by a genetic polymorphism of BDNF, associated with its reduced activity [28]. This observation is pertinent to the present results because it is well known that ketosis increase the expression of BDNF by an epigenetic mechanism involving the KBs histone deacetylase inhibition [33]. Therefore, we propose as a possible neural mechanism by which metabolic ketosis induces an increase in BDNF release which in turn normalizes the basic interictal PREP lack of habituation and prevents migraine.

Another posssible mechanism of cortical activity normalization could be that KD influences the expression of cortical neurotransmitters, by KBs increase in tricarboxylic acid anaplerosis [34], leading to increased brain GABA (the principal inhibitory neurotransmitter) levels in humans [35], and lower levels of neuronal glutamate (the principal excitatory neurotransmitter) in rats [36]. The biosyntesis of both neurotransmitters is connected because glutamate is the precursor of GABA. It is unclear why the majority of glutamate during ketosis follows the pathway of decarboxylation for the GABA biosynthesis, which subsequently modifies the ratio between the two neurotransmitters and creates an increase in overall cortical inhibition. However, we can also not exclude the possibility that anaplerosis leads to an higher biosinthesis of both GABA and glutamate, with the latter that is oxidated for energetic purposes [37] because of the reduction of glucose availability. In addition, regardless of ketone body concentrations, a high-fat diet increases the GABA levels in rat brains [38]. All these mechanisms lead to an increase in GABA concentration both in cells [39, 40] and synaptosomes [41], and the reduction of glutamate at presynaptic level [42]. These changes which modify the GABA/glutamate balance, could account for normalization of cortical PREP, as well as normalization of cortical responses to other sensory modalities [19]. This proposed mechanism is very similar to the one implicated in the antiseizure action of the KD that involves decreased excitatory neurotransmitter release from presynaptic neurons [43, 44]. Additionally, beta-hydroxybutyrate (the most abundant of the three KBs) has been demonstrated to be a direct agonist for the type A receptors of GABA in *Xenopus laevis* oocytes [45], partially accounting for the reduced cortical excitability.

Moreover, it is well known that migraineurs brain shows impairment of oxidative metabolism, mitochondrial functioning, and energetic production [46–48]. Since neuronal excitability depends on energy metabolism, another possible explaination for the KD related cortical excitability normalization is the strengthening of mitochondrial biogenesis, energy metabolism [49], and energy production, by increasing the efficiency of the oxidative respiratory complex [50]. This leads to a more efficient synaptic transmission and neuronal plasticity [51, 52].

On the contrary, nBR is mediated by a polysynaptic neural network mainly modulated by monoaminergic neurotransmitters (especially dopamine and serotonin) involving the spinal trigeminal nucleus, interneurons of the bulbo-pontine lateral reticular formation, and motoneurons of the facial nucleus innervating orbicularis oculi muscles [53, 54]. In a previous paper, we observed that the monoaminergic activity could influence evoked response at electrical nociceptive trigeminal stimulation. In fact, in a patient with cluster headache, we observed a restoration of physiologic habituation of nBR during treatment with a dopamine agonist modulation [55]. Moreover, we observed that the genetic polymorphism of the MAOA (predicting for monoaminergic dysfunctions) was also able to affect the habituation of PREP, potentially by influencing the brainstem processing of stimuli before their projections to cortical structures [29]. The observation of a normalization of evoked responses as recorded at the cortex, but not at the brainstem level, could suggest that KBs activity is not relevant in terms of monoaminergic modulation, limiting its range of action only at the cortical level. Also the energetic improvement due to KD might have a minor role in the brainstem, since its energy requirement is smaller in the brainstem than in the cortex [56].

The modification of cortical response to trigeminal nociceptive stimuli parallels the improvement in migraine clinical features, and is consistent with our previous findings observed recording other cortical EPs [19]. Thus, we further confirm that the normalization of habituation during the KD is a nonspecific response to repetitive stimuli of migraineurs’ cortices due to ketogenesis, irrespective of the modality of stimulation.

There are several potential limitations of this study. The study is not adequately powered to show a statistically significant clinical effect compared to placebo, only by within subject comparison. Only a well-designed randomized clinical trial will demonstrate the effectiveness of KD in prevention of migraine. However, the aim of this study is – consistent with our previous observation [19] – to explore the early neurophysiologic changes induced by a short duration KD and the study was adequately powered to achieve the statistical significance in our observations. Moreover, unlike our previous analogous study [19], to limit possible confounding factors we restricted our observation only in a homogeneous group of patients with migraine without aura that underwent to a normo-caloric diet and did not lose their weight during the month on KD (Table 2). Additionally, we cannot exclude that other mechanisms not related to metabolic ketosis (for instance, lowering the activity of the so-called nutrient-integrating pathways or the lack of fluctuations of glucose and insulin blood levels) [57] could account for the results we have observed. Nonetheless, one could argue that two recordings, the 1st before and the 2nd after KD, could be not enough to verify the potential changes induced through the diet and then we need many more recordings after KD (days or months after KD). However, what we did (recording only ones after the intervention) is perfectly in line with most of the literature using neurophysiology and neuroimaging techniques to investigate mechanisms of action of pharmacological and non-pharmacological interventions [58–68].

Related to the electrophysiological methodologies, 5 per block averaged stimuli could be not enough to measure stable relevant PREP components, although previous papers reliably assessed PREP amplitude and habituation by averaging blocks composed from 3 trails [69]. Finally, we must highlight that there has been some controversy regarding the reliability of PREP evoked by electric stimuli upon that evoked by laser stimuli to test nociception [70]. According to a multichannel scalp recording study, both laser-evoked (LEP) and electrical-concentric trigeminal potentials are similar in amplitude, morphology, and topographic cortical representations, but different in latency, so that Authors conclude that cortical potentials evoked by electric stimulation are contaminated by A-beta non-nociceptive fibre coactivation [70]. Further study using LEPs to assess more selectively pain processing in migraineurs treated with KD is needed in order to confirm our present data.

## Conclusions

In summary, our study confirms that in migraineurs on a ketogenic diet, the typical interictal deficit of habituation of evoked responses to repeated electric painful trigeminal stimuli can normalize, but only at cortical levels (as measured by the recording of PREPs), not in the brainstem (studied by the nBR analysis). These findings suggest that the cerebral cortex may be the primary site of KD-related modulation.

## Data Availability

The datasets used and/or analysed during the current study are available from the corresponding author on reasonable request.

## Abbreviations

BDNF: Brain derived neurotrophic factor
BMI: Body Mass Index
HV: Healthy Volunteers
KB: Ketone Body
KD: Ketogenic Diet
LEP: Laser Evoked Potential
MAD: Modified Atkins Diet
nBR: Nociceptive Blink Reflex
PREP: Pain-Related Evoked Potential
VAS: Visual Analogue Scale
